# Characterization of an Iron-Copper Bimetallic Metal-Organic Framework for Adsorption of Methyl Orange in Aqueous Solution

**DOI:** 10.1155/2023/9985984

**Published:** 2023-08-24

**Authors:** Xiuzhen Yang, Changye Wang, Bin Zhou, Shuangchan Cheng

**Affiliations:** School of Civil Engineering, Hunan University of Science and Technology, Xiangtan 411201, Hunan, China

## Abstract

Iron-based organic frame material MIL-53 (Fe) was synthesized by the hydrothermal method with Cu^2+^ incorporated to obtain bimetallic composite MIL-53 (Fe, Cu). The structure and morphology of the material were characterized by SEM, XRD, BET, FTIR, XPS, and zeta potential. The adsorption performance of MIL-53 (Fe, Cu) on methyl orange was tested under a variety of conditions, including the effects of pH and material dosage, by the static adsorption test. The results show that under the condition of pH = 7, a temperature of 30°C, and an adsorbent dosage of 20 mg, the removal rate of MIL-53 (Fe, Cu) for methyl orange can reach more than 96% within 4 h, and the maximum adsorption capacity after fitting by the thermodynamic model can reach 294.43 mg/g, showing the excellent adsorption performance of MIL-53 (Fe, Cu) on methyl orange. In addition, by exploring the adsorption mechanism of MIL-53 (Fe, Cu) on methyl orange, it is found that the adsorption of MIL-53 (Fe, Cu) on methyl orange depends on chemical adsorption, as evidenced by combining Fe^3+^ and Cu^2+^ in the material with methyl orange molecules to form complexes to achieve adsorption. While the specific surface area of the material had no obvious effect on adsorption, the effects of pH, temperature, and concentration were explored. At a pH of 6.5, greater adsorption occurred at higher temperatures, as determined by thermodynamic model fitting, as well as with higher initial methyl orange molecule concentration.

## 1. Introduction

The rapid development of textile, leather, paper, food, printing, and paint industries has led to water pollution becoming a major threat to many living organisms [1]. Dye wastewater produced by these industries increases the chroma of water, decreases irradiation of water, and poses a serious threat to aquatic life and potentially human health as well [2]. Methyl orange, an azo dye commonly used in printing and textile industries, is a known carcinogen [3], thus making an efficient and economical means of removal from waste water attractive. Adsorption of methyl orange as a possible means to a simple, practical, and renewable [4] remediation method represents an important area of research.

Metal-organic framework materials (MOFs) act as a crystalline porous material with a repeating network structure composed of inorganic metal ions and organic ligands connected through self-assembly [5–7]. MOFs are not only important as research materials in inorganic, organic, and crystal chemistry, but they also display excellent performance in drug delivery [8], gas storage [9], and catalysis due to their unique properties as adsorbents [10]. Compared with ordinary adsorption materials, MOFs have the advantages of a porous structure, large specific surface area, and facile adjustment of pore size [11–13]. These features make the surface binding ability of MOFs robust, resulting in higher adsorption affinity and greater adsorption capacity. Furthermore, MOF surfaces have a high concentration of carboxylic functional groups, which make them ideal for contaminant removal. Pollutants are adsorbed on MOFs through both physical [14] and chemical adsorption [15] through the force of attraction between liquid molecules and the backbone atoms of MOFs as well as the interactions with surface functional groups.

Among the variety of MOFs, MIL-53 (Fe) has the advantages of good structural stability and high density of unsaturated metal nodes [14]. Pollutants in water can be directly absorbed by electrostatically to organic ligands through *π*-*π* interactions and hydrogen bonding [4]. Furthermore, the incorporation of Cu^2+^ in MIL-53 (Fe) can reduce the electron density of the Fe center, reduce the band gap, accelerate the Fe^3+^ and Fe^2+^ cycles, and improve degradation efficiency. Despite these benefits, there are few reports on the effectiveness of Cu^2+^ doping in MIL-53 (Fe).

In this study, Cu^2+^ was successfully incorporated in MIL-53(Fe) to make bimetallic material MIL-53 (Fe, Cu). MIL-53 (Fe, Cu) composites were analyzed by a variety of methods to determine the effects of Cu^2+^ incorporation on morphological characteristics, crystal structure, and functional groups. In addition, the adsorption isotherm model and the adsorption kinetic model were used to determine the adsorption performance of the composite material and explore the mechanism of methyl orange adsorption, which, when combined with the characterization analysis, informs a new direction for adsorption-based remediation of methyl orange.

## 2. Experiment

### 2.1. Reagents and Instruments

The main experimental reagents of this experiment are shown in Table 1.

The main experimental instruments in this experiment are shown in Table 2.

### 2.2. Preparation of Materials

FeCl_3_·6H_2_O (2.027 g), CuCl_2_·2H_2_O (1.279 g), 1,4-H_2_BDC (2.49 g), and DMF (40 mL) were added sequentially to the beaker. After sonication, the contents of each beaker were combined, mixed for 10 min, and transferred to a reactor at 150°C for 17 h. Upon cooling to ambient temperature, the product was washed with deionized water and hot ethanol solution until the supernatant was clear so as to wash away the incomplete material and then collect by centrifugation. The final product was dried under vacuum at 80°C to obtain an orange product, MIL-53 (Fe, Cu).

### 2.3. Adsorption Experiment

The effects of the dosage and pH on methyl orange adsorption by MIL-53 (Fe, Cu) were investigated.

*Influence of the Dosage.* Solutions were prepared as follows: To each of 8 test tubes were added 20 ml of 200 mg/L methyl orange solution and 5, 10, 15, 20, 25, 30, 40, and 50 mg MIL-53 (Fe, Cu), respectively. pH was adjusted to 7 with NaOH and HCl and then placed in a thermostatic shaker at 30°C and 150 r/min. After 4 h, samples were taken after filtration using a 0.45 *μ*m membrane, the concentration of remaining methyl orange was measured at 464 nm using a UV/Vis spectrophotometer.

The effect of pH. Each of 10 test tubes containing 20 ml of 200 mg/L methyl orange solution and 20 mg MIL-53 (Fe, Cu) were adjusted to a final pH of 2, 3, 4, 5, 6, 7, 8, 9, 10 and 11 and placed in a thermostatic shaker at 30°C and 150 r/min. After 4 h, samples were taken after filtration using a 0.45 *μ*m membrane, and the remaining methyl orange concentration was measured at 464 nm using a UV/Vis spectrophotometer.

The adsorption efficiency was evaluated as the removal rate *R* (%) and the adsorption capacity as *q*_*t*_ (mg/g), calculated from (1) and (2), respectively:

(1) $$\begin{array}{c} R=\frac{\left(C_{0}-C_{t}\right)}{C_{0}}\times 100\%\text{,} \end{array}$$

(2) $$\begin{array}{c} q_{t}=\frac{\left(C_{0}-C_{t}\right)\times V}{m}\text{,} \end{array}$$

where *q*_*t*_ is the adsorption amount *t*_min_ of adsorbent methyl orange, mg/g, *C*_0_ is the initial concentration of methyl orange, mg/L, *C*_*t*_ is the methyl orange concentration after *t*_min_, mg/L, m is the dosage of the adsorbent, g, and *V* is the volume of the solution and *L*.

### 2.4. Model Fitting

Adsorption isotherms were fitted to the Langmuir model and the Freundlich model with the following equations:

(3) $$\begin{array}{c} q_{e}=\frac{q_{\max}K_{L}C_{e}}{1+K_{L}C_{e}}\text{,}\\ q_{e}=K_{F}{C_{e}}^{\left(1/n\right)}\text{,} \end{array}$$

where *q*_*e*_ is the adsorption amount of methyl orange at adsorption equilibrium, mg/g, *Q*_max_ is the maximum adsorption capacity, mg/g, *K*_*L*_ is the Langmuir constant, L/mg, *K*_*F*_ is the Freundlich constant, L/mg, 1/*n* is the constant of the affinity of the adsorption reaction, and *C*_*e*_ is the concentration of methyl orange in the solution at adsorption equilibrium, mg/L.

Three groups of methyl orange solution with the concentration gradient (100–500 mg/L) were prepared. pH was adjusted to 6.5, and 20 mg of MIL-53 (Fe, Cu) was added to each group. The groups were then placed into a constant temperature oscillation reactor at 25°C, 35°C, and 45°C for 4 hours, respectively, to ensure that the material was fully adsorbed with methyl orange. Finally, the supernatant was used to measure the remaining methyl orange concentration at a wavelength of 464 nm using a UV/Vis spectrophotometer. The adsorption capacity was calculated, and the Langmuir and Freundlich models were used to fit the experimental data.

The adsorption process data were fitted to quasi-first-order and quasi-second-order kinetic equations as follows:

(4) $$\begin{array}{c} q_{t}=q_{e}\left(1-e^{-k_{1}t}\right)\text{,}\\ q_{t}=q_{e}-\frac{q_{e}}{k_{2}q_{e}t-1}\text{,} \end{array}$$

where *q*_*e*_ is the adsorption amount of methyl orange at adsorption equilibrium, mg/g, *q*_*t*_ is the adsorption amount of methyl orange at *t*_min_, mg/g, *k*_1_ and *k*_2_ are the quasi-first-order and quasi-second-order model rate constants, g/(mg·min), and *t* is the adsorption time, min.

**Figure 1:**
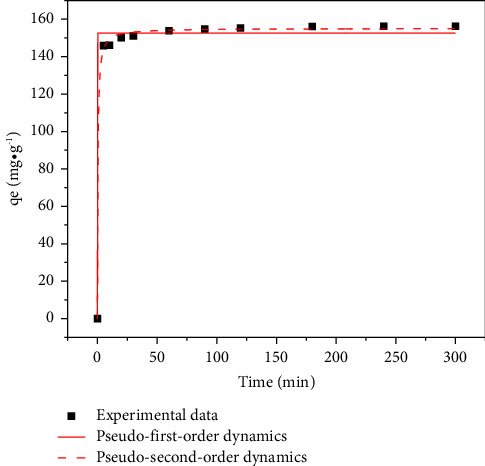
Fitting curves of the pseudo-first-order and pseudo-second-order dynamic models.

To each of ten 20 mL methyl orange solutions at 200 mg/L was added 20 mg MIL-53 (Fe, Cu). The solutions were then reacted in a constant temperature (25°C) oscillation reactor for 5, 10, 15, 20, 30, 60, 90, 120, 180, 240, and 300 min. The remaining methyl orange concentration in the supernatant was determined at a wavelength of 464 nm by using a UV/Vis spectrophotometer. The results, shown in Figure 1, indicate that within 0–5 min, the adsorption capacity of the adsorbent increased rapidly from 5 to 300 min and the adsorption capacity of the adsorbent increased slowly, with the growth rate gradually decreasing before reaching equilibrium.

## 3. Results and Discussion

### 3.1. Material Properties

#### 3.1.1. SEM

**Figure 2:**
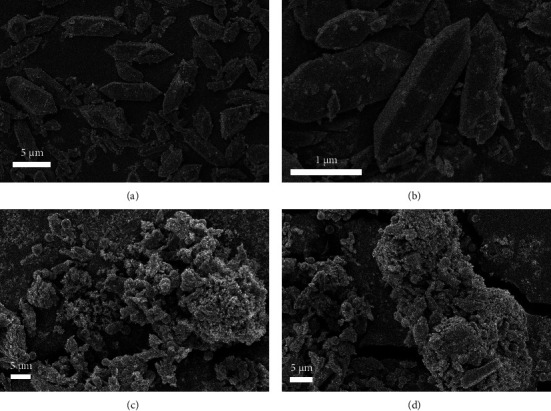
SEM of MIL-53 (Fe, Cu) (a, b) and its adsorption on methyl orange (c, d).

The morphological features of MIL-53 (Fe, Cu) are shown in Figures 2(a) and 2(b). The sample size is relatively uniform, the surface is relatively smooth, and the shape presents a crystalline bar octahedral structure [15, 16]. As shown in Figures 2(c) and 2(d), the morphological characteristics of MIL-53 (Fe, Cu) are indicated after the adsorption of methyl orange. It can be seen that after adsorption of methyl orange, the material is no longer an angular crystalline rod polyhedral structure with multiple bonding, but its morphology has become more disordered and irregular. This indicates that methyl orange has been successfully adsorbed on the surface of the MOF crystals, causing significant morphological changes.

#### 3.1.2. XRD

**Figure 3:**
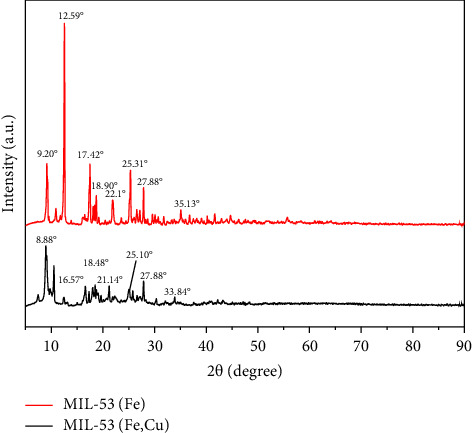
XRD patterns of MIL-53 (Fe) and MIL-53 (Fe, Cu).

The XRD spectra of MIL-53 (Fe) and MIL-53 (Fe, Cu) are shown in Figure 3. The spectra of MIL-53 (Fe) showed diffraction peaks at 2*θ* = 9.2°, 12.59°, 17.42°, and 25.31°, which fit very well with the previously reported MIL-53 (Fe) spectra, indicating the successful synthesis of MIL-53 (Fe) [17]. The difference in the intensity of the peaks and the position of the weak peaks may be attributed to the different reaction times of synthetic MIL-53 (Fe).

In addition, MIL-53 (Fe, Cu) shows two diffraction peaks near 2*θ* = 8.8° compared to MIL-53 (Fe) with three peaks originally at 2*θ* = 17.42°, 18.91°, and 22.1° shifted to 16.57°, 18.48°, and 21.14°, respectively. This may be due to the addition of Cu^2+^ in precursors for MOF synthesis competing for coordination with Fe ions, thus promoting the growth of some crystal surfaces, resulting in a partial change of the crystal structure. At the same time, the peak shape of MIL-53 (Fe, Cu) is smoother with fewer peaks. This may be due to the disordered crystal structure caused by the doping of Cu^2+^ during material synthesis. [18] However, the peak of MIL-53 (Fe, Cu) remained sharp, indicating MIL-53 (Fe, Cu) has a high degree of crystallinity [19].

#### 3.1.3. N_2_ Adsorption-Desorption

**Figure 4:**
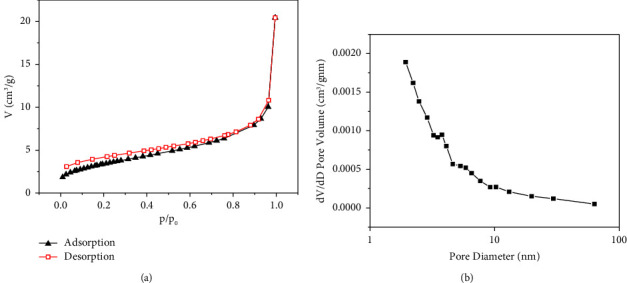
N_2_ adsorption-description isotherms and pore-size distribution curves of MIL-53(Fe, Cu). (a) MIL-53 (Fe, Cu) N_2_ adsorption-desorption of the isotherms. (b) MIL-53 (Fe, Cu) aperture distribution curve.

Figure 4(a) depicts the N_2_ adsorption-desorption isotherm of MIL-53 (Fe, Cu), showing the shape of the type IV isotherm with a H_3_ lag loop, indicating that MIL-53 (Fe, Cu) has slit pores [20–22]. The adsorption material displays higher absorption at higher relative pressure (*P*/*P*_0_), indicating that MIL-53 (Fe, Cu) has a mesoporous structure and belongs to mesoporous material [20–23]. At a relative pressure of 0–0.9, the hysteresis ring corresponds to the slot hole formed by the accumulation of MIL-53 (Fe, Cu) flake particles and the small mesoporous hole formed by the contact between the edge and surface. At a relative pressure of 0.9–1.0, the hysteresis ring corresponds to the larger mesoporous pores formed by the edge-surface contact of MIL-53 (Fe, Cu) flake particles.  Figure 4(b) depicts the pore size distribution curve of MIL-53 (Fe, Cu). As shown in the figure, the pore diameter distribution of MIL-53 (Fe, Cu) is roughly distributed between 3.23 and 10.25 nm, indicating that this material has a highly uniform pore structure [24]. The pore structure parameters of MIL-53 (Fe, Cu) in Table 3 show that the specific surface area of newly prepared MIL-53 (Fe, Cu) is only 12.34 m^2^/g, while the maximum adsorption amount of the material at 45°C was 294.43 mg/g. This indicates that the specific surface area has very little effect on the adsorption of methyl orange, suggesting instead absorbance may be dominated by chemical adsorption. The comparison of different types of MIL-53 and MIL-53 (Fe, Cu) surface area is shown in Table 4. The specific surface area of MIL-53 (Fe, Cu) prepared in this study is lower than that of other types of MIL-53, which may be due to the reduction of the specific surface area caused by the incorporation of Cu^2+^ [29].

#### 3.1.4. FTIR

**Figure 5:**
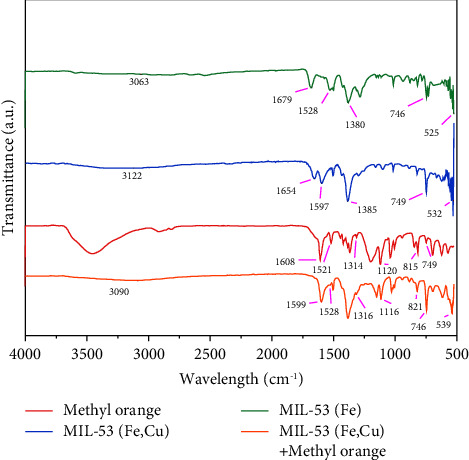
FTIR spectra of MIL-53 (Fe), methyl orange, and MIL-53 (Fe, Cu) before and after adsorption of methyl orange.

Figure 5 shows the infrared spectra of MIL-53 (Fe), methyl orange, and MIL-53 (Fe, Cu) before and after adsorption of methyl orange. As shown, when Cu^2+^ is doped in MIL-53 (Fe), the spectral changes are small indicating that MIL-53 (Fe, Cu) retains the skeleton structure and main functional group composition as original MIL-53 (Fe) [30]. Both MIL-53 (Fe) and MIL-53 (Fe, Cu) have stretching vibration peaks corresponding to C-H and O-H bonds adsorbed on the surface near 3063 cm^−1^ [8, 17, 31], symmetric and asymmetric stretching vibration peaks of -COO- of terephthalic acid appeared at 1528 and 1380 cm^−1^ [32–34], and a tensile Fe-O vibration peak observed at 525 cm^−1^ [35, 36]. Taken together, the above peaks indicate that MIL-53 (Fe, Cu) was successfully synthesized. The characteristic peaks of methyl orange appeared at 749, 815, 1120, 1314, 1521, and 1608 cm^−1^. The characteristic peaks at 749 and 815 cm^−1^ were attributed to the C-H bending vibration of the benzene ring, the characteristic peaks at 1120 and 1314 cm^−1^ were attributed to the symmetric and asymmetric vibrations of the sulfone group, and the characteristic peaks at 1521 and 1608 cm^−1^ were attributed to the aromatic ring C=C telescopic vibration. Comparing the FTIR spectra before and after the adsorption of methyl orange by MIL-53 (Fe, Cu), it can be found that after the adsorption of methyl orange by MIL-53 (Fe, Cu), the characteristic peak intensity around 746, 821, 1528, and 1599 cm^−1^ increased and new characteristic peaks appeared at 1116 and 1316 cm^−1^, which indicated that methyl orange had been successfully adsorbed to MIL-53(Fe, Cu). Furthermore, the disappearance of the absorption peak of C=O at 1654 cm^−1^ and the Fe-O vibration peak at 532 cm^−1^ displayed a blue shift (+7 cm^−1^) after the addition of methyl orange, indicating that C=O and Fe-O are involved in the adsorption process, and finally, the peaks at 800–1200 cm^−1^ became stronger, which may be caused by the complex of Fe^3+^, Cu^2+^, and methyl orange molecules in MIL-53 (Fe, Cu) [4, 37].

#### 3.1.5. XPS

**Figure 6:**
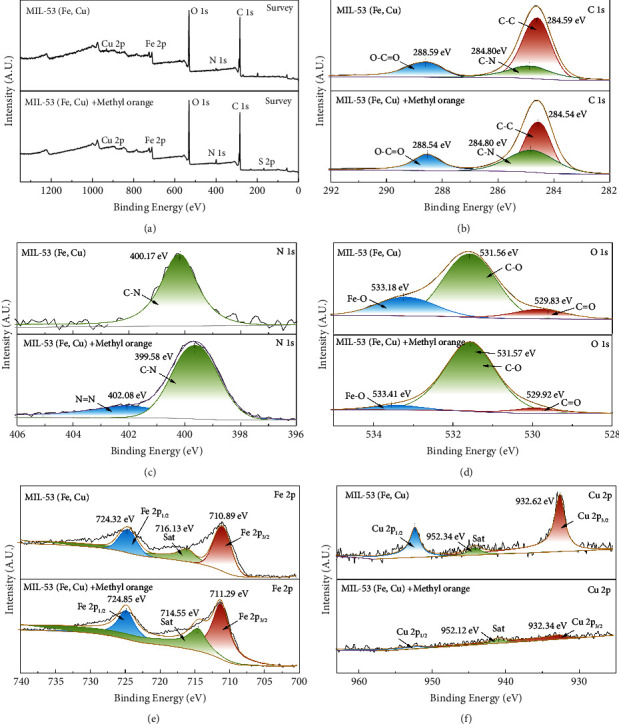
XPS spectra of MIL-53(Fe, Cu) before and after adsorption of methyl orange. (a) Full spectrum map before and after adsorption. (b) C before and after adsorption. (c) N before adsorption. (d) O after adsorption. (e) Fe before and after adsorption. (f) Cu before and after adsorption.

The element composition and chemical states of the MIL-53 (Fe, Cu) surface before and after adsorption were analyzed by XPS. As seen from the measurement curve in Figure 6(a), C, N, O, Fe, and Cu are present in MIL-53 (Fe, Cu), while the characteristic spectrum of S appears after adsorption of methyl orange. In addition, the percentage of N increases from 0.35% to 0.96%, consistent with successful adsorption of methyl orange on MIL-53 (Fe, Cu) [38, 39].

As shown in Figure 6(b), the C 1 s spectrum of MIL-53 (Fe, Cu) corresponds to the C-C, C-N, and O-C=O bonds at 284.54 eV, 284.80 eV, and 288.59 eV, respectively [39, 40]. After the adsorption of methyl orange, the peaks of C–C and O-C=O shift to 284.54 eV and 288.54 eV, respectively, which is possibly due to the electronic interaction between the elements in the adsorption process [41]. The N 1 s spectrum in Figure 6(c) has only one peak at a binding energy of 400.17 eV, corresponding to the C-N bond [42]. After adsorption of methyl orange, a new N=N bond was observed at a binding energy of 402.08 eV and the C-N peak was also shifted to 399.58 eV [15, 17, 38, 43]. At the same time, the content of the C-N bond also increased relative to before methyl orange adsorption. The above observations may be explained by the introduction of dye molecules [44]. The O 1 s spectrum is decomposed into three peaks, located at 529.83 eV, 531.56 eV, and 533.18 eV, corresponding to C=O, C-O, and Fe-O bonds, respectively [17, 45]. During methyl orange adsorption, the C=O, C-O, and Fe-O peaks all shifted toward higher binding energies, indicating a decrease in the outer electron density of oxygen in the organic ligand [39]. Moreover, the area of C=O and Fe-O binding peaks changed significantly after methyl orange adsorption, indicating that C=O and Fe-O functional groups are involved in the adsorption process.

Figure 6(e) shows Fe 2p_3/2_ and Fe 2p_1/2_ in Fe^3+^ peaks at 710.89 eV and 724.32 eV, respectively [46]. The distance between the two peaks, 13.43 eV, is very similar to *α*-Fe_2_O_3_ [15]. In addition, a satellite peak was observed at 716.13 eV, indicating the presence of Fe^3+^ in MIL-53 (Fe, Cu) [47].  Figure 6(f) shows two peaks of the Cu 2p spectrum at 932.62 eV and 952.34 eV, corresponding to Cu^2+^ 2p_3/2_ and Cu^2+^ 2p_1/2_, respectively. The other peak in the 932.62 EV–952.34 eV range is the satellite peak of Cu^2+^ [48].

#### 3.1.6. Zeta Potential

**Figure 7:**
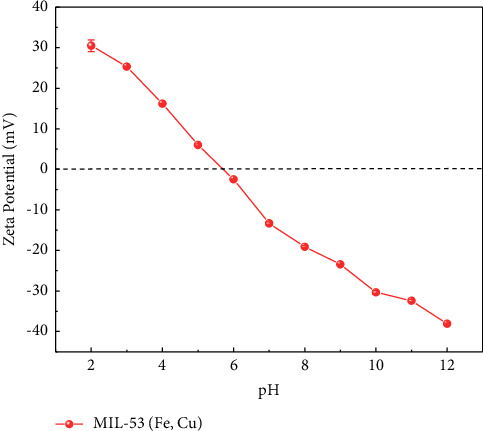
Zeta potential curve with pH.

The zeta potentials of MIL-53 (Fe, Cu), recorded at different pH values, are shown in Figure 7. As seen in the figure, the pH_PZC_ (zero charge point) of the material is 5.3. When solution pH is greater than 5.3, the adsorbent surface is negatively charged, and when solution pH is less than 5.3, the adsorbent surface is positively charged. Methyl orange, an anionic azo dye, exists as a quinoid structure under acidic conditions and an azo structure under basic conditions. Regardless of whether the solution is acidic or alkaline, methyl orange is anionic and negatively charged in an aqueous solution.

Taken together, this means that the electrostatic attraction between negatively charged methyl orange and the cation on the adsorbent surface leads to an increased adsorption capacity when the pH of the solution is less than 5.3.

## 4. Adsorption Properties

### 4.1. Univariate Experiment

#### 4.1.1. Effect of the MIL-53 (Fe, Cu) Dosage

**Figure 8:**
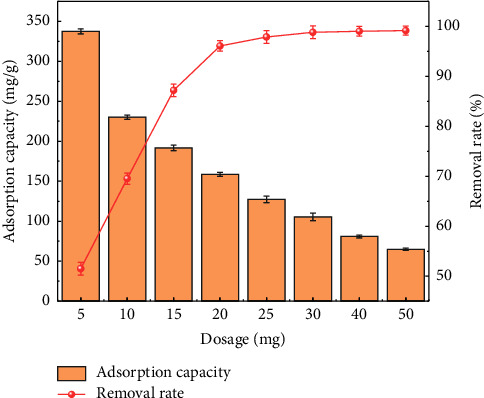
Effect of the dosage on adsorption of methyl orange by MIL-53 (Fe, Cu).

Figure 8 shows that when all other factors are constant, the removal rate of methyl orange by MIL-53 (Fe, Cu) increases with an increasing dosage, while at the same time, the adsorption capacity decreases with an increasing dosage. It is speculated that the increased dosage results in increased adsorption vacancy points carried by the adsorbent. This also increases the adsorption sites that can accommodate methyl orange, leading to an increased removal rate. When the concentration and volume of methyl orange solution are held constant, while the dosage of the adsorbent is increased, the total adsorption sites carried by the material also increase, resulting in a reduction in the percentage of the total adsorption sites occupied by methyl orange and a decreased absorption capacity. In summary, the dosage effect studies show 20 mg to be the best dosage of the adsorbent.

#### 4.1.2. Effect of pH

**Figure 9:**
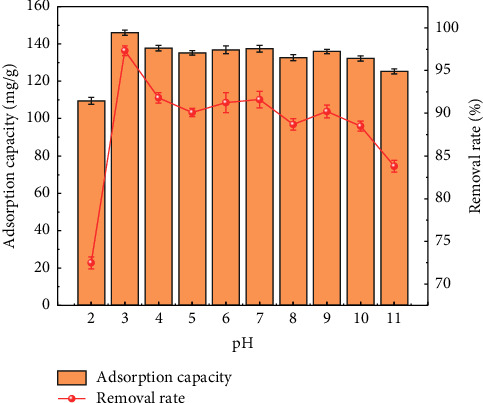
Effect of pH on adsorption of methyl orange by MIL-53 (Fe, Cu).

Solution pH is a key factor affecting the adsorption performance. The resulting adsorption efficiencies are shown in Figure 9. When pH = 3, the removal rate and adsorption capacity were the highest, reaching 97.37% and 146.22 mg/g, respectively. With the pH increasing from 3 to 11, the removal rate and adsorption capacity decreased to 84.40% and 126.74 mg/g, respectively. The effect of pH on the adsorption performance is due to the electrostatic interaction between the adsorbent and dye molecules. Given the pH_PZC_ of MIL-53 (Fe, Cu) of 5.3, when solution pH is greater than 5.3, the adsorbent is negatively charged, which is not conducive to the adsorption of negatively charged methyl orange. When solution pH is less than 5.3, the adsorbent is positively charged and the negatively charged methyl orange is attracted electrostatically. At higher pH values, hydroxide also hindered the adsorption of methyl orange on the surface of MIL-53 (Fe, Cu), resulting in a decreased removal rate with increasing pH. However, when solution pH was 2, the removal rate and adsorption capacity were only 72.60% and 109.03 mg/g, respectively. This may be because methyl orange is largely in the form of R-SO_4_H when solution pH is 2. The presence of high concentration of H^+^ not only inhibits the formation of molecular aggregates but also weakens the interaction between molecules and adsorbent particles, thereby increasing the distance between molecules and adsorbent particles, resulting in a lower adsorption efficiency.

### 4.2. Adsorption Isotherms

**Figure 10:**
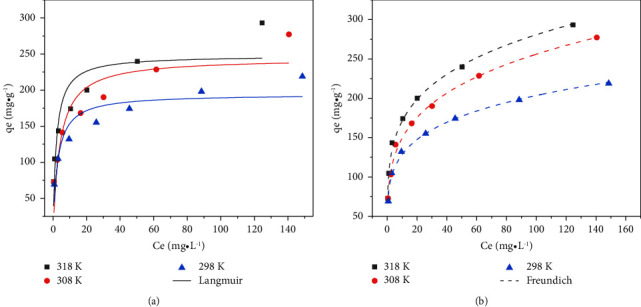
Sorption isotherm fitting curve: (a) Fitting curve of the Langmuir model. (b) Fitting curve of the Freundlich model.

The fitted nonlinear adsorption isotherms and calculated parameters are shown in Figure 10 and Table 5, respectively.

As can be seen from Table 5, the correlation coefficient (*R*^2^) of the Freundlich model is significantly higher than that of the Langmuir model under the three temperature gradients, indicating that the Freundlich model is more consistent with the experimental data than the Langmuir isotherm model. Meanwhile, the maximum adsorption capacity calculated by the Freundlich model is 294.43 mg/g, which is close to the experimentally determined value of 293.25 mg/g. The good agreement of the experimental data with the Freundlich isotherm model indicates the nonuniform distribution of surface active sites and the nonsingle interaction among adsorbents.

### 4.3. Thermodynamic Parameters

In this study, the thermodynamic data were fitted, and the Gibbs free energy (Δ*G*), enthalpy (Δ*H*), and entropy changes (Δ*S*) of the adsorption reaction were calculated as follows:

(5) $$\begin{array}{c} K_{D}=\frac{q_{e}}{C_{e}}\text{,} \end{array}$$

(6) $$\begin{array}{c} ∆G=∆H-T∆S\text{,} \end{array}$$

with Δ*H* and Δ*S* derived from the following equation:

(7) $$\begin{array}{c} \ln K_{D}=\frac{∆S}{R}-\frac{∆H}{RT}\text{,} \end{array}$$

where *q*_*e*_ is the amount of methyl orange adsorbed at adsorption equilibrium, mg/g, *C*_*e*_ is the residual concentration of methyl orange at adsorption equilibrium, mg/L, *K*_*D*_ is the temperature equilibrium constant, *R* is the ideal gas constant, generally 8.314 J·mol^−1^·K^−1^, and *T* is the Kelvin temperature, K.

**Figure 11:**
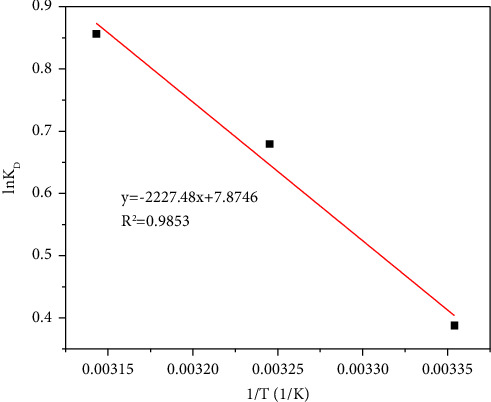
Thermodynamic equation linear fitting curve.

According to the results in Figure 11, the correlation coefficient (*R*^2^) of adsorption for methyl orange is 0.9853, showing a linear relationship. Values for Δ*S* and Δ*H* were calculated according to (7), from the intercept and the slope of the line, and Δ*G*, at the corresponding temperature, was calculated by (6). The calculated results, from Table 6, are as follows: Δ*G*°=°−2.32∼−1.00°<°0, Δ*S*°>°0, and Δ*H*°>°0 under the three temperature gradients in this study. This indicates that the adsorption reaction is a spontaneous, endothermic process with increased disorder in the solid-liquid system [49–51]. Second, the Δ*H* of complexation is between 8 and 60 kJ/mol, while Δ*H* of MIL-53 (Fe, Cu) is 18.52 kJ/mol, which indicates that adsorption is driven by complexation, with the main route occurring by chemoadsorption rather than physical adsorption [13].

### 4.4. Adsorption Dynamics

To understand the adsorption mechanism and possible rate-limiting steps, kinetic analysis was performed. The fitted nonlinear adsorption kinetics and calculation parameters are shown in Figure 1 and Table 7, respectively.

The results show that for MIL-53 (Fe, Cu), *R*^2^ (0.998) of the quasi-second-order kinetic model is greater than that of the quasi-first-order kinetic model (0.990). This indicates that the adsorption of methyl orange is more consistent with a pseudo-second-order kinetic model and that methyl orange is adsorbed on the surface of MIL-53 (Fe, Cu) mainly through chemical interaction [52, 53].

### 4.5. Adsorption Mechanism

The effect of pH on methyl orange adsorption along with zeta potential characterization, which determined pH_PZC_ of MIL-53 (Fe, Cu) to be 5.3, is consistent with a positively charged adsorbent surface interacting electrostatically with anionic methyl orange in the pH range of 2–5.3. The adsorption process is greatly affected by the adsorbent and the adsorbate surface potential, supporting electrostatic interaction as one of the adsorption mechanisms. Furthermore, the results of FTIR and XPS spectra before and after adsorption show that C=O and Fe-O in MIL-53 (Fe, Cu) are involved in the adsorption process and that the protonation/deprotonation of oxygen containing functional groups is implicated in the adsorption of methyl orange. Fe^3+^ and Cu^2+^ in the adsorbent material combine with methyl orange to form a complex. BET characterization analysis showed that although the incorporation of Cu^2+^ resulted in a decrease in a specific surface area, making it lower than other types of MIL-53. However, the incorporation of Cu^2+^ improves the electron utilization efficiency and codecomposition of MO from H_2_O_2_ and indirectly increases the adsorption capacity of MO. Second, from the characterization analysis of SEM and FITR, it can be seen that the incorporation of Cu^2+^ does not change the structure of MIL-53 (Fe) and that the flexible structure of MIL-53 (Fe) is conducive to the adsorption of methyl orange. Finally, the adsorption data are consistent with the Freundlich equation, as well as the pseudo-second-order kinetic model. This lends support to adsorption which occurs mainly through chemisorption means on multilayer heterogeneous surfaces, and physical adsorption is less. Therefore, although the specific surface area of MIL-53 (Fe, Cu) obtained in this study is low, it still has good adsorption performance for methyl orange.

## 5. Reusability of Sorbents

**Figure 12:**
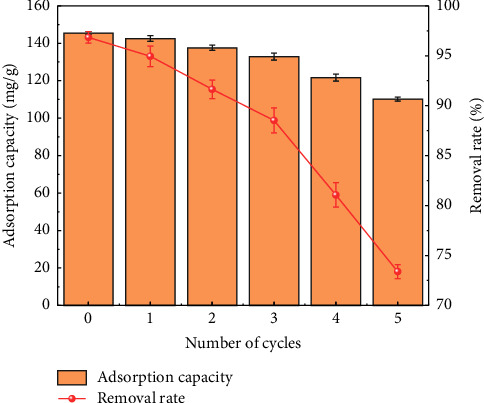
Recovery of MIL-53 (Fe, Cu) adsorption of methyl orange.

A good sorbent should be able to be recycled in practical applications. After the adsorption experiment, MIL-53 (Fe, Cu) after adsorbing methyl orange was soaked in absolute ethanol to elute methyl orange and then collected by centrifugation and dried in a vacuum drying oven. The obtained product was subjected to the adsorption experiment again, the temperature was 30°C, the initial concentration of methyl orange was 200 mg/L, the pH was adjusted to 3, the adsorbent dosage was 20 mg, the adsorption time was 4 h, the adsorption-desorption cycle experiment was performed 5 times, and the experiment was repeated three times.  Figure 12 shows the removal performance of MIL-53 (Fe, Cu) on methyl orange after 5 cycles. The results showed that the removal rate of MIL-53 (Fe, Cu) was more than 70% after 5 adsorption-desorption cycles, indicating that MIL-53 (Fe, Cu) had repeatability and broad application prospects in the removal of methyl orange wastewater.

## 6. Conclusion

Cu^2+^ incorporation partially changed the crystal structure of MIL-53 (Fe) but had no obvious effect on the functional groups of MIL-53 (Fe). Newly prepared MIL-53 (Fe, Cu) is a mesoporous material with slit pores.

Although the specific surface area and pore volume of MIL-53 (Fe, Cu) are not high, it has a high adsorption capacity for methyl orange due to its flexible structure, which may be due to the small physical adsorption effect of the material on methyl orange, and adsorption is mainly chemical adsorption. C=O and Fe-O in MIL-53 (Fe, Cu) participated in the adsorption process, and Fe^3+^ and Cu^2+^ in MIL-53 (Fe, Cu) combined with methyl orange molecules to form a complex, leading to adsorption. The pH_PZC_ of MIL-53 (Fe, Cu) is 5.3. When pH is less than 5.3, the adsorption capacity is increased by electrostatic attraction of negatively charged methyl orange molecules. Adsorption kinetic models indicate that within 0–5 min, the adsorption capacity of the adsorbent increased rapidly from 5 to 300 min and the adsorption capacity of the adsorbent increased slowly, with the growth rate gradually decreasing before reaching equilibrium. Furthermore, the adsorption process conforms to the pseudo-second-order kinetic model (*R*^2^ ≥ 0.995) and the Freundlich equation (*R*^2^ ≥ 0.99), indicating that adsorption is mainly chemical adsorption on a multilayer heterogeneous surface. Thermodynamic parameter analysis shows that adsorption is a spontaneous, endothermic, and entropy-increasing process, and increasing the temperature is beneficial to the adsorption of methyl orange.

## Figures and Tables

**Table 1:** List of the main raw materials and chemical reagents.

Material/reagent name	Purity	Factory
Terephthalic acid (C_8_H_6_O_4_)	AR	Sinopharm Chemical Reagent limited corporation
Ferric chloride hexahydrate	AR	Sinopharm Chemical Reagent limited corporation
N, N-Dimethylformamide	AR	Sinopharm Chemical Reagent limited corporation
Copper chloride dihydrate	AR	Sinopharm Chemical Reagent limited corporation
Absolute ethanol	AR	Hunan Huihong Reagent Co., Ltd
Methyl orange	AR	Macklin
Sodium hydroxide (NaOH)	AR	Aladdin
Concentrated hydrochloric acid (HCl)	AR	Gold Wall Reagent

**Table 2:** List of laboratory equipment.

Instrument name	Model	Manufacturer
Ultrapure water machine	UPH	Xi'an Youpu instrument Equipment Company
Reactor	100 ml	Shanghai Yuming Instrument Equipment Co., Ltd
Electronic balances	JJ224BC	Changshu ShuangJie Testing Instrument Factory
Thermostatic water bath oscillator	THZ-82	Changzhou Guohua Electric Appliance Company
Constant temperature blast drying oven	GZX-9070 MBE	Shanghai Boxun Industrial Company
Vacuum drying oven	DZF-1B	Beijing Kewei Yongxing Instrument Company
Ultrasonic cleaner	KQ5200DB	Kunshan Ultrasound Instrument Company
High-speed centrifuge	TGL16M	Hunan Kaida Scientific Instrument Company
pH meter	PB-16	Sartorius Scientific Instruments
Flame atomic absorption spectrometer	AA-7002A	Beijing Sanxiong Technology Company
UV/Vis spectrophotometer	TU-1901	Beijing General Instrument Co., Ltd

**Table 3:** Pore structure parameters of MIL-53 (Fe, Cu).

Textural parameters	MIL-53 (Fe, Cu)
Surface area (m^2^/g)	12.34
Average pore diameter (nm)	13.63
Micropore volume (cm^3^/g)	0.0026
Total pore volume (cm^3^/g)	0.0316

**Table 4:** Compare surface area of different samples.

Samples	Surface area (m^2^/g)	Reference
MIL-53 (Fe)	35.9	[25]
MIL-53 (Al)	1270	[26]
MIL-53 (Cr)	988	[27]
MIL-53 (Cu)	1150	[28]
MIL-53 (Fe, Cu)	12.34	This article

**Table 5:** Parameters of the isothermal fitting models.

*T* (K)	Langmuir			Freundlich		
	*q*_max_	*K*_*L*_	*R* ^2^	*K*_*F*_	1/*n*	*R* ^2^
298.15	194.343	0.376	0.854	81.038	0.200	0.994
308.15	244.316	0.249	0.837	86.982	0.234	0.995
318.15	248.025	0.518	0.831	104.107	0.215	0.994

**Table 6:** Thermodynamic parameters of the methyl orange adsorption process.

*T* (K)	Δ*S* (kJ/mol)	Δ*H* (kJ/mol)	Δ*G* (kJ/mol)
298.15	0.0655	18.52	−1.00
308.15			−1.66
318.15			−2.32

**Table 7:** Dynamic model parameter values.

Pseudo-first-order kinetic constants		Pseudo-second-order kinetic constants	
*K*_1_	*R* ^2^	*K*_2_	*R* ^2^
1455765	0.990	0.016	0.998

## Data Availability

The data that support the findings of this study are available from the corresponding author on request.
